# Molecular Mapping of *D_1_*, *D_2_* and *ms5* Revealed Linkage between the Cotyledon Color Locus *D_2_* and the Male-Sterile Locus *ms5* in Soybean

**DOI:** 10.3390/plants2030441

**Published:** 2013-07-05

**Authors:** Alina Ott, Yang Yang, Madan Bhattacharyya, Harry T. Horner, Reid G. Palmer, Devinder Sandhu

**Affiliations:** 1 Department of Biology, University of Wisconsin-Stevens Point, Stevens Point, WI 54481, USA; 2 Department of Agronomy, Iowa State University, Ames, IA 50011, USA; 3 Department of Genetics, Development and Cell Biology, Ames, IA 50011, USA; 4 Microscopy and NanoImaging Facility, Iowa State University, Ames, IA 50011, USA

**Keywords:** *Glycine max*, genetic linkage mapping, cotyledon color, hybrid seed, male sterility, tapetum disintegration

## Abstract

In soybean, genic male sterility can be utilized as a tool to develop hybrid seed. Several male-sterile, female-fertile mutants have been identified in soybean. The male-sterile, female-fertile *ms5* mutant was selected after fast neutron irradiation. Male-sterility due to *ms5* was associated with the “stay-green” cotyledon color mutation*.* The cotyledon color trait in soybean is controlled by two loci, *D_1_* and *D_2_.* Association between cotyledon color and male-sterility can be instrumental in early phenotypic selection of sterility for hybrid seed production. The use of such selection methods saves time, money, and space, as fewer seeds need to be planted and screened for sterility. The objectives of this study were to compare anther development between male-fertile and male-sterile plants, to investigate the possible linkages among the *Ms5*, *D_1_* and *D_2_* loci, and to determine if any of the *d_1_* or *d_2_* mutations can be applied in hybrid seed production. The cytological analysis during anther development displayed optically clear, disintegrating microspores and enlarged, engorged pollen in the male-sterile, female-fertile *ms5ms5* plants, a common characteristic of male-sterile mutants. The *D_1_* locus was mapped to molecular linkage group (MLG) D1a and was flanked by Satt408 and BARCSOYSSR_01_1622. The *ms5* and *D_2_* loci were mapped to MLG B1 with a genetic distance ~12.8 cM between them. These results suggest that use of the *d_2_* mutant in the selection of male-sterile line may attenuate the cost hybrid seed production in soybean.

## 1. Introduction

Mutations that affect microsporogenesis and microgametogenesis leading to male sterility have been described for many plant species [1]. Several meiotic mutations have been identified in soybean (*Glycine max* (L.) Merrill) [2]. These include mutations affecting male and female meiosis (*st* mutants), mutations that affect only male development and cause male-sterility by cytoplasmic genes (*cms*) or nuclear genes (*ms* mutants), and mutations that affect only the female and cause female partial sterility (*fsp* mutants) [2].

Manual cross-pollination for the production of large quantities of hybrid soybean seeds can be difficult and time consuming. Male-sterile mutants are considered a powerful tool in hybrid breeding programs. A cytoplasmic male sterility (CMS) system is the ideal for hybrid seed production [1]. Several *cms* systems have been reported in soybean, and are being studied for their potential in hybrid seed production [3,4,5,6,7].

Alternatively, several methods have been proposed to use nuclear genic male-sterile plants to produce hybrid seed [8,9]. Genic male sterility in soybean is used to improve efficiency of hybridization by eliminating tedious hand emasculations, to enhance random mating for population development [10,11,12] and recurrent selection [13], and to facilitate the development of testers for inbred line evaluation and development [12,14]. So far, in soybean, eleven nuclear male-sterile, female-fertile mutants have been reported [2,15].

There are several reports of DNA markers linked to male-sterile, female-fertile genes in soybean. The Midwest Oilseed male-sterile (*msMOS*) was positioned on MLG D1b of the USDA-ARS-ISU map and segregated as a single recessive Mendelian locus [15]. Male-sterile, female-fertile mutant *ms3* (T284) was also positioned on MLG D1b [16], in the same chromosomal region as *msMOS*, and female-partial sterile I (*FspI*) mutant [17]. The *ms2* and *ms9* mutants were positioned on MLG O and MLG N, respectively [16]. Two additional, environmentally sensitive male-sterile, female-fertile mutants, *msp* and *ms8*, that showed variable fertility based on the night temperature, were mapped to MLG D1b and M, respectively [18]. All male-sterile, female-fertile genes are non-allelic. 

Recent soybean heterosis studies revealed 42% increased seed yield over that of the mean parent yield [19,20,21]. Positive heterosis values for seed yield from cytoplasmic male-sterile systems have been reported in a recent International Conference on Utilization of Heterosis in Crops [22,23,24,25]. Recent discoveries of nuclear male-sterile and cytoplasmic-genetic male-sterile systems have enhanced the feasibility of F_1_ hybrids in commercial soybean production. In the final analysis, the success of F_1_ hybrid soybean will require the efficient transfer of fertile pollen from the male parents to the male-sterile, female-fertile parents. As soybean flower morphology is adapted for self-pollination, a well-developed functional nectary [26], together with a suitable insect pollen vector system, is a practical solution for F_1_ seed production in soybean. Insect-mediated cross-pollination has been improved through phenotypic recurrent selection for enhancing hybrid seed production in soybean [14,27]. 

The *ms5* male-sterile, female-fertile mutant (T277) was the result of fast neutron irradiation [28]. The mutant is inherited as a single-gene recessive trait; however, no cytological studies have been conducted. The cotyledon color trait in soybean is controlled by two loci: *D_1_* and *D_2_* [29,30]. Carter and Burton [31] discovered an association between the green cotyledon trait and the *ms5* locus. Due to the distinctive nature of the cotyledon color phenotype, this association can play an important role in screening for true hybrid seed. A male-sterile line that had the green cotyledon trait could be used as female parent with any yellow-seeded male parent. Selfed seeds on the green cotyledon female parent will be green. However, hybrid seeds from pollination with pollen from yellow cotyledon plants will be yellow. Thus F_1_ hybrid seeds can be identified prior to planting [32]. Cooper and Tew [33] demonstrated use of the linkage between *ms5* and green cotyledon, to produce seed homozygous for male sterility, which may become instrumental in facilitating hybrid seed production in soybean [2]. The objectives of this study were to compare anther development between male-fertile and male-sterile plants, to investigate the possible linkages among the *Ms5*, *D_1_* and *D_2_* loci and to determine if any of the *d1* or *d2* mutations can be applied in hybrid seed production.

## 2. Materials and Methods

### 2.1. Microscopy: ms5

Near-anthesis anthers were collected in Carnoy’s fixative and then stained with iodine-potassium iodide to identify both filled and empty pollen grains [34]. Stained anthers were squashed between a slide and coverslip to expose the pollen for bright-field microscopy.

Both *Ms5Ms5* and *ms5ms5* anthers were dissected from very young buds through open flowers to obtain the full range of microsporogenesis (from sporogenous mass stage to late microspore stage) and microgametogenesis (young to dehiscing pollen). Whole anthers at all stages were immediately immersed in fixative at room temperature (RT), consisting of 2% glutaraldehyde and 2% paraformaldehyde in a 0.1 M cacodylate buffer (v/v), pH 7.24, and stored at 4 °C for 48 h [35]. Anthers were thoroughly rinsed in 3 changes of cold buffer for 30 min each, followed by a secondary fixative of 1% osmium tetroxide in the same buffer at room temperature for 4 h. Anthers were washed several times in deionized water for a total of 1 h and then placed in a 2% aqueous solution of uranyl acetate (bulk staining) for 4 h. Anthers were rinsed in deionized water for about 1 h and then subjected to an ascending acetone dehydration series (10%, 20%, 30%, 50%, 70%, 90%, 100%, 100% ultrapure). Spurr’s resin (hard) [36] infiltration steps of 5:1 (acetone:resin mixture), 3:1, 1;1, 1:3, and 1:5 were carried out over several days and were followed by 2 changes of pure resin mixture before flat casting and polymerization at 60 °C for 2 d [35]. 

Anthers were cut out of the casts and oriented to produce cross sections. One-micrometer-thick sections were cut with a diamond knife using a Leica Ultracut S Ultramicrotome, placed on droplets of deionized water on glass slides and dried down on a warming tray. Sections were stained with a dilute solution of toluidine blue O and dried. Permount mounting medium was added along with a coverslip.

Anther sections and pollen grain squashes were viewed with either an Olympus BH10 or a Zeiss Axioplan II light microscope in the bright-field mode and imaged using 20× and 40× objectives and images were captured with an attached Zeiss MrC digital camera on each microscope. Captured black and white images were processed in CSS Adobe PhotoShop and Illustrator for display. 

### 2.2. Mapping Population: ms5

A segregating F_2_ population derived from self-pollination of F_1_ seed was developed from the cross of cultivar Manchu (PI 30593; *Ms5Ms5*) × T277H, *Ms5ms5*. Fertile plants from segregating progeny of T277H were used as male parents. Manchu was used as the female parent.

During the summer of 2005, F_2_ plants of the cross Manchu × T277H were grown at the Bruner Farm near Ames, Iowa. About 220 seeds of each F_2_ family were planted with 15 cm between plants within a row. At the beginning of flowering, stage R1 [37], flowers of 10 plants per F_2_ family were collected to identify *ms5* segregation by the presence of aborted pollen grains. On that basis, one segregating F_2_ family was selected as the mapping population, which consisted of 103 plants (Table 1). At maturity, each F_2_ plant was phenotypically scored as either fertile or sterile. The fertile plants were single-plant threshed.

**Table 1:** F_2_ and F_2:3_ segregation patterns, Chi-square and P-values for populations of Manchu (*Ms5Ms5*) × T277H (*Ms5ms5*).

Population	No. F_2_ plants				No. F_2:3_ families			
	Fertile	Sterile	χ^2^ (3:1)	*P*	All fertile	Segregating	χ^2^ (1:2)	*P*
**A12-g-8A**	76	27	0.08	0.78	25	51	0.01	0.94

### 2.3. Progeny Testing: ms5

The F_3_ seeds from individual F_2_ fertile plants were harvested, and seeds planted in the summer of 2006 at the Bruner Farm. Segregation for fertility/sterility in the F_2_-derived progeny rows was used to determine the genotype of each fertile F_2_ plant. The expected phenotypic ratio in the F_2_ generation was 3:1 (male-fertile:male-sterile plants), and the expected genotypic ratio of the F_2:3_ population was 1:2:1 (*Ms5Ms5*:*Ms5ms5*:*ms5ms5*).

### 2.4. Mapping Population: d_1_d_2_

Segregating F_2_ populations were derived from self-pollination of three F_1_ plants and were developed from the cross of cultivar Minsoy (*D_1_D_1_D_2_D_2_*; PI 27890) × cultivar Harosoy isoline for green cotyledon (*d_1_d_1_d_2_d_2_; L69-4267*) (Table 2). Minsoy was used as the female parent.

During the summer of 2009, F_2_ plants of the cross Minsoy × Harosoy (*d_1_d_1_d_2_d_2_*) were grown at the Bruner Farm near Ames, Iowa.

**Table 2:** F_2_ and F_2:3_ segregation patterns for cotyledon color, Chi-square and *P*-values for three populations of Minsoy (*D_1_D_1_D_2_D_2_*; PI 27890) × Harosoy (*d_1_d_1_d_2_d_2_*; L69-4267).

Population	No. F_2_ plants		χ^2^ (15:1)	*P*	No. F_2_ families				χ^2^ (7:4:4:1)	*P*
	Yellow	Green			All yellow	15:1	3:1	All green		
**A09-308**	118	5	1	0.32	61	31	26	5	2.64	0.45
**A09-309**	118	7	0.01	0.76	48	26	44	7	6.99	0.07
**A09-310**	117	9	0.17	0.68	55	29	33	9	0.43	0.94
**Total**	353	21	1.18	0.28	164	86	103	21	1.81	0.61

### 2.5. Progeny Testing: d_1_d_2_

F_2_ and F_2:3_ seed was phenotypically scored in the field. The expected segregation ratio of 15:1 (yellow:green) was observed for the F_2_ seed. The expected segregation ratio of 7:4:4:1 (*D_1__D_2__*, *D_1_D_1___ or __D_2_D_2_*:*D_1_d_1_D_2_d_2_*:*D_1_d_1_d_2_d_2_ or d_1_d_1_D_2_d_2_*:*d_1_d_1_d_2_d_2_*) was observed for the F_2:3_ seed (Table 2). The *d_1_d_1_d_2_d_2_* (green cotyledon color) genotype was true breeding green cotyledon color. 

For progeny scoring, the families that were showing all green plants in F_2:3_ progeny test were scored as “B”. The families that segregated 3:1 in F_2:3_ were scored as “C”: either *D_1_d_1_* was heterozygous and *d_2_d_2_* was homozygous or *d_1_d_1_* was homozygous and *D_2_d_2_* was heterozygous. The F_2:3_ families that segregated 15:1, indicating *D_1_d_1_* and *D_2_d_2_*, both were heterozygous, were scored as “H”. The F_2_ plants (*D_1_D_1_D_2_D_2_*, *D_1_D_1_D_2_d_2_*, *D_1_D_1_d_2_d_2_*, *D_1_d_1_D_2_D_2_*, *d_1_d_1_D_2_D_2_*) that did not segregate for yellow and green cotyledon color in the F_2:3_ generation were not used for mapping analysis. 

### 2.6. Molecular Analysis

Two young leaves were collected from each parent and F_2_ plants of the *Ms5* and *D_1_D_2_* mapping populations. A CTAB protocol for DNA extractions described in previous research was followed [38]. The initial SSR screening was done using the bulked segregant analysis (BSA) method [39]. Two green cotyledon bulks were prepared using DNA from 10 green cotyledon plants each. DNA bulks were prepared by pooling 0.5 μg DNA from each of the selected F_2_ plants. Each bulk was diluted to a final concentration of 50 ng DNA/μL. A total of 600 simple sequence repeat (SSR) markers from the 20 soybean chromosomes were used (Supplemental Table S1). The markers showing polymorphisms between Minsoy and green cotyledon bulks were used to screen 210 selected F_2_ plants. Additional markers in the regions showing segregation were also used to screen individuals.

A similar method was used for *ms5*. Two bulks, consisting of 10 male-sterile plants each, were prepared. The markers showing polymorphisms between Manchu and the two male-sterile bulks were used to screen 103 F_2_ plants. Additional polymorphic markers in the region were also used to screen individuals.

Sequence information for developing SSR markers was obtained from Song *et al*. (2004) [40] and SoyBase [41]. For SSR analysis, 30 ng DNA was used as the template in a 10 μL reaction containing 1× reaction buffer (10 mM Tris-HCl, 50 mM KCl, pH 8.3), 2.0 mM MgCl_2_; 0.25 μM of each primer; 200 μM of each dNTP and 0.25 units of *Biolase* DNA polymerase (Bioline, USA Inc., Taunton, MA, USA). The PCR conditions were as follows: 2 min at 94 °C; 35 cycles of 30 s at 94 °C, 30 s at 47 °C, 1 min at 72 °C; followed by 8 min at 72 °C. The amplification products were separated on a 4% agarose gel and the DNA products were photographed using UV light.

### 2.7. Data Analysis

Each plant in the F_2_ population was scored according to its SSR alleles at the locus, for Minsoy or Manchu depending upon the F_2_ population, *i.e*., a score of “A” was assigned if the plant was homozygous for the alleles from Minsoy or Manchu, “B” if it was homozygous for the alleles from mutant, and “H” if it was heterozygous. After scoring the population, recombination values were calculated to determine if each given SSR marker was linked to the gene of interest. Mapmaker V3.0 was used to make the final map that included all linked SSR markers [42]. A minimum logarithm of the odd ratio (LOD) score of 3 was used for accepting linkage between two markers. Recombination frequencies were converted to map distances in cM using the Kosambi map function [43].

## 3. Results

### 3.1. Cytological Analyses

**Figure 1:**
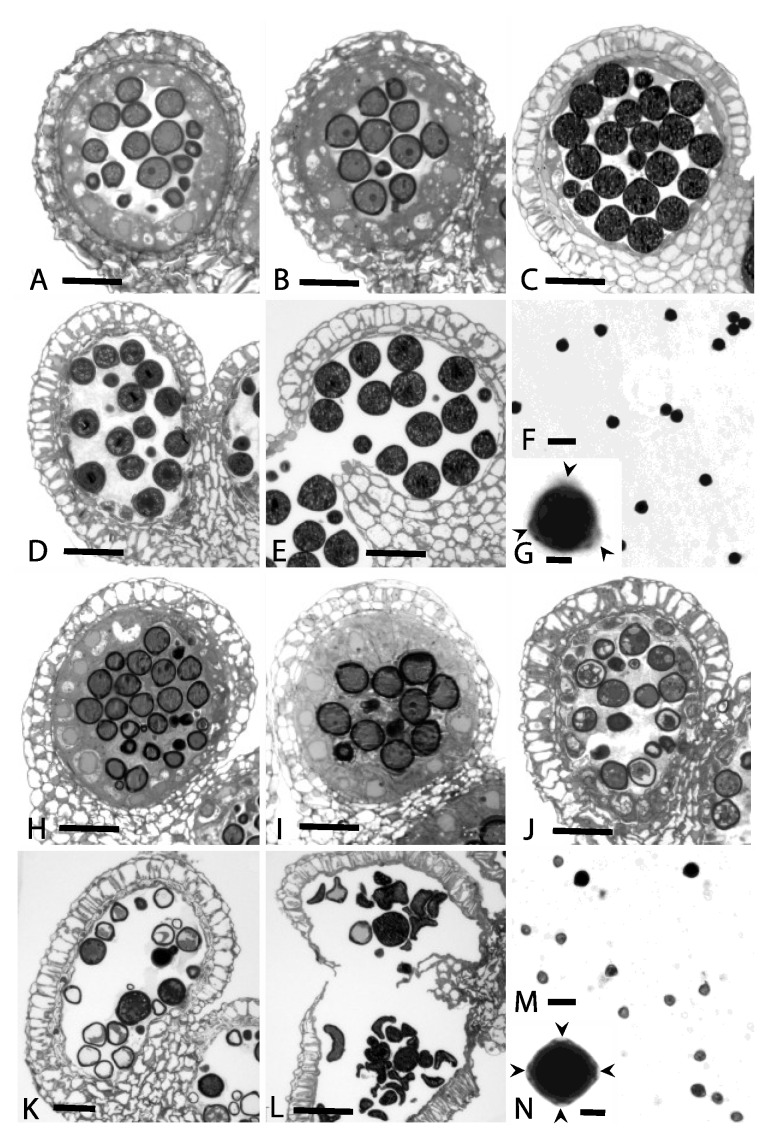
One-μm thick cross sections through male-fertile (MF = A–E) and *ms5ms5* (ms = H–L) anthers depicting stages from early-mid microspores through pollen dehiscence, and anther squashes showing both normal (MF = F and G) and aborted or abnormal (ms = M and N) microspores/pollen from both lines. (**A**) MF = mid-microspore stage with tapetum; (**B**) MF = late-microspore stage with tapetum; (**C**) MF = pollen stage with disintegrating tapetum; (**D**) MF = pollen stage with disintegrated pollen; (**E**) MF = late pollen stage near dehiscence. Adjacent locules have fused; (**F**) MF = anther squash showing viable, stained pollen grains; (**G**) MF = single tricolporate pollen grain with three arrowheads pointing to pore regions; (**H**) ms = mid-microspore stage with tapetum; (**I**) ms = mid- to late-microspore stage with tapetum; (**J**) ms = late-microspore/early pollen stages showing both aborting microspores and enlarged pollen; (**K**) ms = late-microspore stage with many aborted microspores, an enlarged pollen, and a disintegrated tapetum; (**L**) ms = disintegrated and collapsed microspores and pollen from two adjacent, fused locules. Tapetum is absent and anther wall has split open; (**M**) ms = anther squash showing non-staining, non-viable microspores and several abnormal, engorged enlarged pollen; (**N**) ms = single, engorged pollen with four arrowheads pointing to four colpi. Scale bars = 40 μm on A-E, H-L; 50 μm on F and M; and 25 μm on G and N.

A microscopic study was conducted to compare the anther development at various stages in male-fertile (*Ms5Ms5*) versus male-sterile (*ms5ms5*) floral buds. The male-fertile (MF) line goes through both normal microsporogenesis (Figure 1A,B) and microgametogenesis (Figure 1C–G) ending with dehiscence and pollen release. This normal development contrasts with the *ms5ms5* (ms) line (Figure 1H–N) that begins to show abnormal development at the late-microspore/early pollen stage (Figure 1J) with disintegrating microspores and enlarged pollen (male cells). The tapetum surrounding the male cells at this stage is vacuolate and disintegrating. At a slightly later stage (Figure 1K) more vacuolated microspores and enlarged pollen are visible. The tapetum is disintegrated. Near the time of dehiscence (Figure 1L), both collapsed microspores and enlarged pollen are visible in the locules.

The male-fertile line squashes show heavily stained, engorged pollen. These pollen grains are tricolporate (Figure 1G, arrowheads). In contrast, the *ms5ms5* squashes show many degenerated and optically clear microspores and enlarged, engorged pollen (Figure 1M). Some of the enlarged, stained pollen display four distinct colpi (Figure 1N, arrowheads).

### 3.2. Progeny Testing: ms5

The F_2_ and F_2:3_ populations from the Manchu (*Ms5Ms5*) × *Ms5ms5* cross showed monogenic inheritance of the *ms5* gene. Self-pollination of heterozygous F_1_ plants from the crosses of Manchu (*Ms5Ms5*) × *Ms5ms5* segregated 3 fertile:1 sterile plant in the F_2_ generation (Table 1). Each fertile F_2_ plant, generated from the heterozygous F_1_ plant, was threshed individually and progenies of each plant were tested for segregation into sterility and fertility phenotypes. The F_2:3_ family segregation fit the expected 1 homozygous fertile:2 heterozygous ratio (Table 1).

### 3.3. Molecular Mapping of ms5

**Figure 2:**
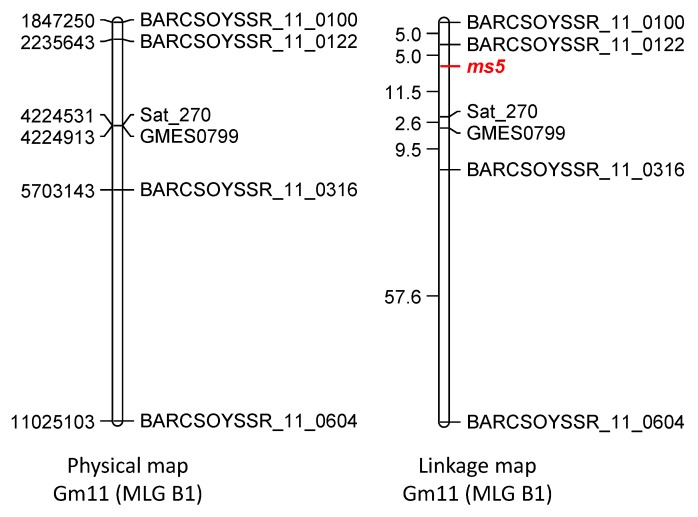
Genetic linkage mapping of the *ms5* gene from the cross Manchu (PI 30593) × T277H; *Ms5ms5*. Genetic and physical maps of soybean chromosome Gm11 (MLG B1) showing location of the *ms5* locus. Genetic distances are shown in centiMorgans (cM) and physical distances are shown in base pairs (bp).

BSA was used to map the *ms5* gene using 600 SSR markers representing all 20 MLGs (Supplementary Table S1). Sat_270, located on MLG B1, showed putative linkage to *ms5*. An additional 20 markers from MLG B1 were tested for polymorphisms between parents. Only six markers (BARCSOYSSR_11_0100, BARCSOYSSR_11_0122, Sat_270, GMES0799, BARCSOYSSR_11_0316, and BARCSOYSSR_11_0604) were polymorphic. Analysis of these six polymorphic markers on 103 individual F_2_ plants allowed generation of a genetic linkage map of the *ms5* region. The *ms5* locus was positioned between BARCSOYSSR_11_0122 and Sat_270, with the closest marker BARCSOYSSR_11_0122 mapped at a 5.0 cM distance from *ms5* (Figure 2).

### 3.4. Molecular Mapping of D_1_ and D_2_

The χ^2^ calculations for the *d_1_d_1_d_2_d_2_*-segregating population showed a good fit to the genotypic ratio of 15 yellow cotyledon:1 green cotyledon in all three F_2_ populations. Segregation of the F_2:3_ generation showed a good fit of 7:4:4:1 homozygous yellow cotyledon:segregating 15 yellow to 1 green cotyledon: Segregating 3 yellow to 1 green cotyledon:all homozygous green cotyledon ratio (Table 2). These data confirm duplicate dominance epistasis between *D_1_* and *D_2_*.

**Figure 3:**
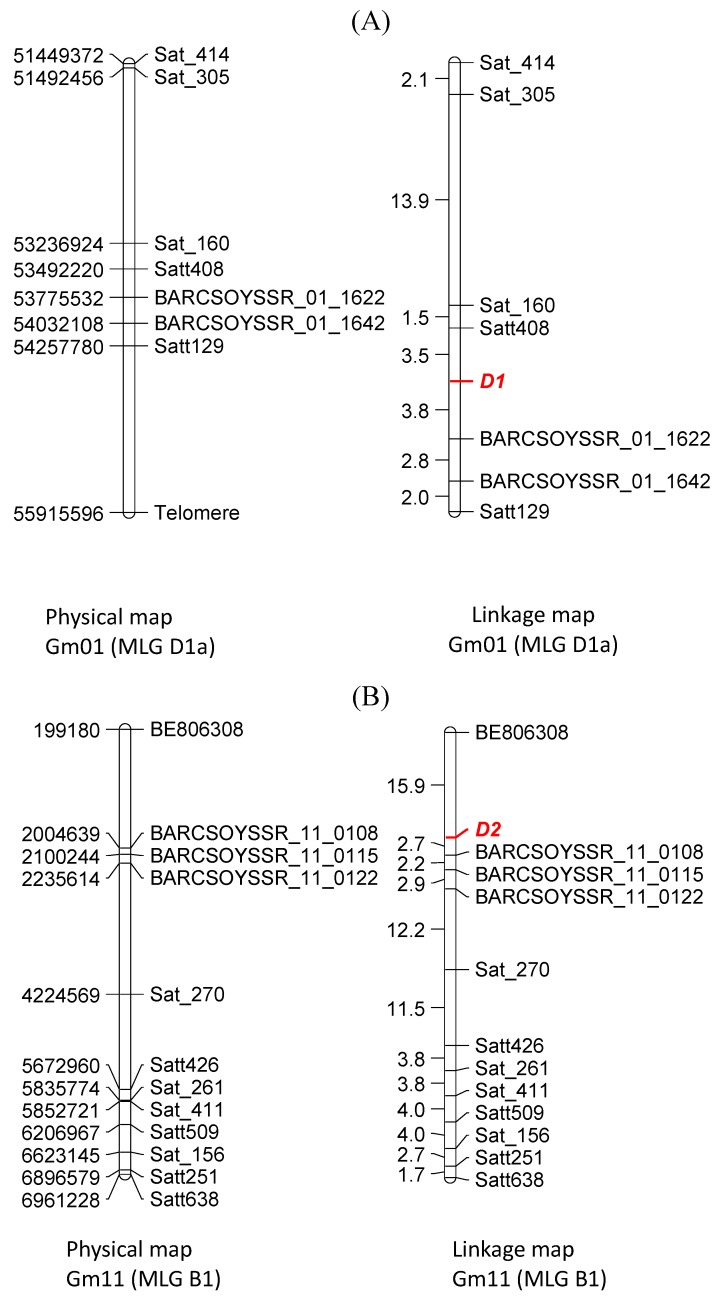
Genetic linkage mapping of cotyledon color genes from the cross Minsoy (*D_1_D_1_D_2_D_2_*; PI 27890) × Harosoy (*d_1_d_1_d_2_d_2_*; L69-4267). (**A**) Genetic and physical maps of soybean chromosome Gm01 (MLG D1a) showing location of the *D1* locus. (**B**) Genetic and physical maps of soybean chromosome Gm11 (MLG B1) showing location of the *D2* locus. Genetic distances are shown in centiMorgans (cM) and physical distances are shown in base pairs (bp).

Initial BSA results using Minsoy and the two green cotyledon bulks indicated linkage of cotyledon colorwith Satt129 and Sat_270*.* Satt129 is located on Gm01 (MLG D1a) and Sat_270 is located on Gm11 (MLG B1) [40]. A previous study showed that the *D1* locus is linked with seed coat color gene G that is mapped to MLG D1a [30]. Twenty-one SSR markers on MLG D1a (chromosome Gm01) were tested on the parents for polymorphism, and seven (Satt129, BARCSOYSSR_01_1622, BARCSOYSSR_01_1642, Satt408, Sat_160, Sat_305, and Sat_414) detected polymorphism. Thirty-three markers on chromosome Gm11 were tested on the parents for polymorphism, and twelve (BE806308, BARCSOYSSR_11_0108, BARCSOYSSR_11_0115, BARCSOYSSR_11_0122, Satt251, Satt426, Satt509, Satt638, Sat_156, Sat_261, Sat_270, and Sat_411) showed polymorphism. Polymorphic markers were used to assay DNA of 210 individual F_2_ plants that segregated 15:1 or 3:1 or were all green (Figure 3). The map for Gm01 showed that the *D_1_* locus was flanked by Satt408 and BARCSOYSSR_01_1622 (Figure 3A). Mapping results for Gm11 revealed the location of *D_2_* between BARCSOYSSR_11_0108 and BE806208 on MLG B1 (Figure 3B).

By using the known physical locations of the markers, putative gene candidates were explored [44]. The 283 Kb interval around *D_1_* contains 33 annotated genes. The 1.8 Mb interval around D_2_ contains 243 annotated genes.

## 4. Discussion

### 4.1. Cytological Analyses

Male-fertile, female-fertile *Ms5Ms5* and *Ms5ms5* plants displayed normal microsporogenesis and microgametogenesis, as expected. Sterile plants, *ms5ms5*, showed abnormal development at the late-microspore/early pollen stage, typical of many male-sterile mutants [1]. Another characteristic of many male-sterile plants is tapetum disintegration, which was observed in *ms5ms5* plants [1]. Aborted pollen grains displayed various degrees of staining with I_2_KI and shapes. Four distinct colpi were observed occasionally and are characteristic of 2n pollen from tetraploid (80 chromosome) soybean plants (Figure 1N) [45]). Occasionally, outcrossed seed was observed on field-grown sterile plants. The few seed observed may indicate some degree of female sterility [28], lower levels of insect-pollinator attraction, or both. Megasporogenesis and megagametogenesis were not studied. 

A previous investigation suggested association between cotyledon color (either *D_1_* or *D_2_*) and *Ms5* [30,31]. In this study we mapped *Ms5* to ~2 Mb region on MLG B1 (Figure 2). The close linkage between *Ms5* and one of the genes for green cotyledon color is explained as *D_2_* also mapped to MLG B1 (Figure 3B). One marker in particular, BARCSOYSSR_11_0122, showed linkage to both *Ms5* and *D_2_*. *D_2_* mapped distally to BARCSOYSSR_11_0122 while *Ms5* mapped proximally to the same marker. Based on their distances from BARCSOYSSR_11_0122, *D_2_* and *Ms5* are estimated to be approximately 12.8 cM apart, indicating fair linkage. *D_1_* was mapped to a 283 kb region on MLG D1a (Figure 3A). 

The mutations *d_1_* and *d_2_* affect cotyledon color by the prevention of thylakoid protein degradation, thus retaining the green color in the double mutant *d_1_d_1_d_2_d_2_* [46]. Previous studies have not succeeded in determining the exact gene products of the *D_1_* and *D_2_* genes, though cytokinin or auxin regulation may be involved [47]. The region where *d_2_* mapped contains three cytochrome-related genes, which may be putative gene candidates. However, the region where *d_1_* mapped contains no genes with annotated functions directly related to these pathways.

Linkages between traits of interest and morphological markers have been used with great success in plant breeding, particularly in wheat. In Australian wheat breeding, stem rust resistance conferred by the recessive *Sr2* gene was selected for by screening for pseudo-black chaff [48]. Two other morphological traits, glume color and leaf-tip necrosis, also were used to select for rust resistance [48]. Morphological markers also were used to track chromosomal introgression during backcrossing [49].

One of the main limitations of morphological markers is the developmental stage at which they appear [50]. Seed color or cotyledon color, however, can be used to score for fertility before the seeds are planted, thus allowing for very early screening. In addition, the visual scoring method is simple and inexpensive.

Continued mapping efforts for easily scored phenotypic traits, such as color, provide important resources for the research and breeding community. In particular, seed traits that can be scored before planting have the potential to save valuable land resources and growing time. The linkage between *D_2_* and *Ms5* is especially useful, because the green cotyledon phenotype encoded by the *d_1_d_1_d_2_d_2_* genotype will particularly be suitable in identifying putative male-sterile plants for generating F_1_ hybrids. Although identification of male-sterile plants based on green cotyledons will not be perfect, growing only green cotyledon seeds will significantly increase the recovery of the male-sterile plants. Furthermore, use of pollen donors with yellow cotyledons will facilitate identifying true F_1_ seeds from the male-sterile female parents following insect pollinations. The study also set the stage for molecular cloning of the *Ms5*, *D_1_*, and *D_2_* genes to broaden understanding the mechanisms of male fertility and regulation of chlorophyll biogenesis, respectively.

## 5. Conclusions

Characterization of pollen from sterile *ms5ms5* plants showed tapetum degradation and abnormal development at the late-microspore/early pollen stage, in contrast to normal microsporogenesis and microgametogenesis. Using SSR markers, the male-sterile mutant, *ms5*, and two cotyledon color mutants*, d_1_* and *d_2_*, were mapped to MLG B1, D1a and B1 respectively. Linkage between *Ms5* and *D_2_* can facilitate identification of male-sterile plants for hybrid seed production based on green cotyledons. Knowing the genetic position of these genes will assist in the molecular cloning of these genes. 

## Abbreviations

SSR: Simple Sequence Repeat
PCR: Polymerase Chain Reaction
BSA: Bulked Segregant Analysis
cM: centiMorgan
MLG: Molecular Linkage Group

## Supplementary Material

**Table S1:** Chromosomal distribution of microsatellite markers tested for Bulked Segregant Analysis.

Molecular Linkage Group	Chromosome Number	Number of markers
A1	5	35
A2	8	35
B1	11	25
B2	14	28
C1	4	20
C2	6	23
D1a	1	46
D1b	2	54
D2	17	21
E	15	23
F	13	39
G	18	45
H	12	22
I	20	28
J	16	20
K	9	25
L	19	24
M	7	37
N	3	25
O	10	25
TOTAL		600
